# Alternative splicing after gene duplication drives CEACAM1-paralog diversification in the horse

**DOI:** 10.1186/s12862-018-1145-x

**Published:** 2018-03-15

**Authors:** Sophie Mißbach, Denis Aleksic, Lisa Blaschke, Timm Hassemer, Kyung Jin Lee, Martin Mansfeld, Jana Hänske, Johannes Handler, Robert Kammerer

**Affiliations:** 1 Institute of Immunology, Friedrich-Loeffler-Institut, Suedufer 10, Greifswald, Insel Riems Germany; 2 Plattform Degenerative Erkrankungen, Deutsches Primatenzentrum GmbH, Goettingen, Germany; 3 Department of Cellular Biochemistry, Max-Planck-Institute of Biochemistry, Martinsried, Germany; 4 Department of Chemistry, Korea Advanced Institute of Science and Technology (KAIST), Daejeon, Republic of Korea; 5 Clinic for Horses, Veterinary Faculty, Freie Universität Berlin, Oertzenweg 19b, D-14163 Berlin, Germany; 6 Friedrich-Loeffler-Institut, Bundesforschungsinstitut für Tiergesundheit, Federal Research Institute for Animal Health, Südufer 10, D, 17493 Greifswald, Insel Riems Germany

**Keywords:** Alternative splicing, Gene duplication, Horse, CEA gene family, CEACAM, Signaling motifs, Evolution

## Abstract

**Background:**

The *CEA* gene family is one of the most rapidly evolving gene families in the human genome. The founder gene of the family is thought to be an ancestor of the inhibitory immune checkpoint molecule CEACAM1. Comprehensive analyses of mammalian genomes showed that the *CEA* gene family is subject to tremendous gene family expansion and contraction events in different mammalian species. While in some species (e.g. rabbits) less than three CEACAM1 related genes exist, were in others (certain microbat species) up to 100 CEACAM1 paralogs identified. We have recently reported that the horse has also an extended CEA gene family. Since mechanisms of gene family expansion and diversification are not well understood we aimed to analyze the equine CEA gene family in detail.

**Results:**

We found that the equine CEA gene family contains 17 functional *CEACAM1*-related genes. Nine of them were secreted molecules and eight CEACAMs contain transmembrane and cytoplasmic domain exons, the latter being in the focus of the present report. Only one (*CEACAM41*) gene has exons coding for activating signaling motifs all other *CEACAM1* paralogs contain cytoplasmic exons similar to that of the inhibitory receptor CEACAM1. However, cloning of cDNAs showed that only one *CEACAM1* paralog contain functional immunoreceptor tyrosine-based inhibitory motifs in its cytoplasmic tail. Three receptors have acquired a stop codon in the transmembrane domain and two have lost their inhibitory motifs due to alternative splicing events. In addition, alternative splicing eliminated the transmembrane exon sequence of the putative activating receptor, rendering it to a secreted molecule. Transfection of eukaryotic cells with FLAG-tagged alternatively spliced CEACAMs indicates that they can be expressed in vivo. Thus detection of CEACAM41 mRNA in activated PBMC suggests that CEACAM41 is secreted by lymphoid cells upon activation.

**Conclusions:**

The results of our study demonstrate that alternative splicing after gene duplication is a potent mechanism to accelerate functional diversification of the equine CEA gene family members. This potent mechanism has created novel CEACAM receptors with unique signaling capacities and secreted CEACAMs which potentially enables equine lymphoid cells to control distantly located immune cells.

## Background

The Carcinoembryonic Antigen (CEA)-related cell adhesion molecule 1 (CEACAM1) is a multifunctional cell surface molecule of the immunoglobulin super family (ISF) involved in cell-cell adhesion, vascular remodeling, insulin resistance and immune responses. Two main splice forms of CEACAM1 concerning its cytoplasmic tail were identified. Isoforms with the long cytoplasmic tail provide inhibitory signals via immunoreceptor tyrosine-based inhibitory motifs (ITIM = S/I/V/LxYxxI/V/L) and immunoreceptor tyrosine-based switch motifs (ITSM = TxYxxIV), while the short isoforms do not contain ITIMs/ITSM. Expression of the long isoform dominates in immune cells and the short isoform in epithelial cells, respectively [1, 2]. Various pathogens recruit CEACAM1 as a cellular receptor to invade their hosts and at the same time modify the immune response [3–6]. There is growing evidence that an early hallmark of *CEA* gene family expansion was the generation of paired receptors by creating a CEACAM1 paralog which has a very similar ligand-binding domain but transduce contrary, i.e. activating, signals into the cell as a countermeasure to pathogen attacks [7, 8]. Once such a receptor pair was created, further expansion of the gene family is a critical process, since it may lead to an imbalance of paired receptor signaling. Most likely further diversification of the membrane anchorage and the signaling capacity was a prerequisite for further gene family expansion. Indeed, despite the sometimes tremendous expansion of the CEA gene families, the most populous families are found in certain bat species, containing up to 100 CEACAMs [9], the number of ITIM bearing CEACAMs in one species seem to be strictly limited. In most species only one CEACAM exist which contains ITIM in its cytoplasmic tail. Exceptions of this rule, described so far are mice and xenopus tropicalis, which have two CEACAMs with ITIM motifs http://www.carcinoembryonic-antigen.de/mouse/index.html [8]. The two different ITIM bearing CEACAMs in mice, i.e. CEACAM1 and CEACAM2 have a different expression pattern, excluding a simple duplication of the inhibitory signals in a given cell type [10]. In addition further expansion of the CEA gene family in mice took place by duplication of CEACAMs lacking transmembrane and cytoplasmic domain exons. Although there is an amplification of the inhibitory receptor in humans, none of the paralogous genes encode a functional ITIM [11]. This was achieved by rendering the transmembrane domain of CEACAM1 into a signal peptide for a glycosylphosphatidylinositol (GPI) anchor. For this modification only minimal mutations are required including the introduction of a stop codon in the transmembrane domain exon [12]. No amplification of the inhibitory receptor takes place in the dog genome, but an amplification of activating CEACAMs was observed [13, 14]. Hence, efficient mechanisms must exist for the diversification of signaling capacities, if a duplication of inhibitory receptors is envisaged. We have recently reported that convergent evolution within the CEA gene families of humans and the horse had led to a similar expansion of secreted pregnancy-specific glycoproteins (PSG), which are a subgroup of *CEACAM1* paralogous genes [15–17]. Obviously, secreted CEACAMs, which do not contain a transmembrane and cytoplasmic domain do not transmit signals into the cell and therefore are functionally different from the ancestral inhibitory receptor CEACAM1. Now we have analyzed the evolution of equine *CEACAM* genes containing a transmembrane domain exon and focused on mechanisms of their putative functional diversification. We observed that the expansion of the equine *CEA* gene family is due to the amplification of inhibitory CEACAM receptor genes. Characterization of the transcribed equine *CEACAM1* paralog mRNAs revealed that only two CEACAMs have an ITIM motif and that alternative splicing (AS) after gene duplication (GD) is an important mechanism for functional diversification of duplicated membrane anchored *CEACAMs* in the horse.

## Results

### Comparison of equine CEACAM transmembrane domains

**Fig. 1:**
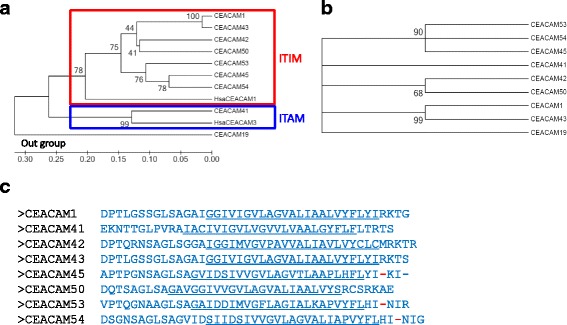
Comparison of equine CEACAM TM domains. Phylogenetic trees were constructed from transmembrane domain exon nucleotide sequences (**a**), and amino acid sequences (**b**) using the UPGMA (**a**) and ML (**b**) method (MEGA 6.0 software). The reliability of a phylogenetic tree was assessed using the Bootstrap test applying 500 replicates. The statistical support for selected nodes is shown. Boxes group CEACAMs with the indicated properties. Note that on amino acid level the association of the TM with cytoplasmic signaling motifs is not visible. The transmembrane domain of Equine *CEACAM19* was used as an out group. **c** Amino acid sequences of transmembrane domains of equine CEACAM-1 related CEACAMs. Predicted sequence of transmembrane helices by the TMHMM Server v. 2.0 http://www.cbs.dtu.dk/services/TMHMM-2.0/ are underlined. Stop codons within the transmembrane domain exon are indicated in red. Amino acids are depicted in single letter code. TM, transmembrane domain; CC, CEACAM; ITAM, immunoreceptor tyrosine-based activation motif; ITIM, immunoreceptor tyrosine-based inhibition motif

In the horse genome, *CEACAM1* and seven *CEACAM1* paralogs exist, which contain exons coding for transmembrane domains [15]. Phylogenetic analyses using nucleotide sequences of equine *CEACAM* transmembrane (TM) domain exons and of human *CEACAM1* and *CEACAM3*, respectively, resulted in trees that comprise two deep clades, one containing human *CEACAM1* TM sequence and 6 closely related equine TM sequences and the other containing human *CEACAM3* TM sequence and a single equine TM sequence of *CEACAM41* (Fig. 1a). Using the amino acid sequences for phylogenetic analyses, the CEACAM1 related TM could be separated into three subgroups (Fig. 1b). Subgroup 1 contains CEACAM1 and CEACAM43, the second subgroup consists of CEACAM42 and CEACAM50, and the third subgroup is composed of CEACAM45, CEACAM53 and CEACAM54. The latter three transmembrane domain exons are harboring a stop codon. For all predicted transmembrane domains complete transmembrane helices were predicted by the TMHMM Server, and arguing against the presence of GPI-anchored CEACAMs in the horse. Indeed, analysis of the sequences for the presence of GPI anchors using the PredGPI Server did not provide any hint for a GPI linkage.

### Expression of putatively membrane-anchored equine CEACAMs

As previously described, we have designed gene specific primer pairs of which the forward primer was located in the leader exon and the reverse primer in the N domain exon, respectively [15]. Using these primers, we performed a comprehensive analysis of gene expression in a set of horse tissues and cells (Tables 1 and 2). Various parts of the horse intestine were hot spots of membrane-bound CEACAM expression. In addition, in the mucosa of the vulva expression of several membrane-bound CEACAMs was found. CEACAM1, CEACAM43 and CEACAM50 were expressed in the liver. CEACAM42 and CEACAM43 were expressed in the kidney. Surprisingly, only CEACAM45 was found to be expressed in the spleen and no expression could be shown for CEACAM41 in tissues analyzed. Since several CEACAMs in humans are considered to play a pivotal role in the immune system, we further investigated CEACAM expression in white blood cells either unstimulated or after the stimulation with 500 U/ml IL-2. As shown in Table 2 granulocytes expressed CEACAM1, CEACAM41 and CEACAM43 while unstimulated PBMC expressed only CEACAM45. However, upon stimulation with IL-2, PBMC were found to express in addition to CEACAM45, also CEACAM1, CEACAM43 and CEACAM41 (Table 2).

**Table 1:** Expression of membrane anchored equine CEACAMs in various tissues of the horse

Tissue	CC1	CC19	CC41	CC42	CC43	CC45	CC50	CC53	CC54
Salivary gland									+
Tongue		++							++
Oesophagus		++						++	+
Liver	+++				++		+		
Duodenum	++			++	+++	+++	++		++
Jejunum				+	++	++			
Ileum				+++	++	++	+++		++
Caecum	+			++	+	+++	++		+++
Colon				++	+	++	+		++
Rectum	+			++	+	++			
Trachea								++	++
Lung					+				
Kidney				+	++				
Bladder									
Vulva	+			+		+++		++	+++
Spleen						+			

number of animals = 2

+++ = strong expression; ++ = moderate expression; + = weak expression

**Table 2:** Expression of membrane anchored equine CEACAMs in naïve and IL-2 stimulated immune cells

Cell type	CC1	CC19	CC41	CC42	CC43	CC45	CC50	CC53	CC54
PBMC						+			
PBMC 7d_IL2	++		++	+	+++	++			
PBMC 14d_IL2			++		+	++			
PBMC 19d_IL2			++		+	+++			
Granulocytes	+++		+^a^		++				

*n* = 3

+++ = strong expression; ++ = moderate expression; + = weak expression

^a^only in one sample positive

### Alternative splicing (AS) of equine *CEACAM* mRNAs

**Fig. 2:**
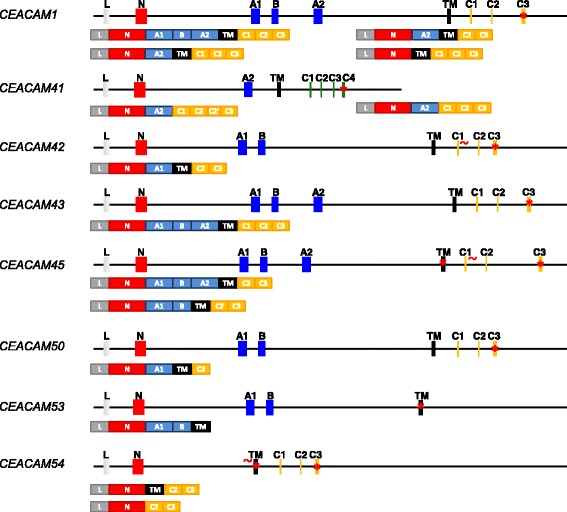
mRNA of equine *CEACAMs*. From all indicated *CEACAMs* the genomic exon structure is shown on top and below the different transcript variants observed in this study were depicted. Differential splicing was found for *CEACAM1* (4 transcripts), *CEACAM41*, *CEACAM45* and *CEACAM54* (each 2 different transcripts). Transcripts coding for secreted CEACAMs were found for *CEACAM41* and *CEACAM54*. No transcript coding for a CEACAM with an ITAM signaling motif was identified. Transcripts coding for CEACAMs with ITIM/ITSM motifs were found only for two *CEACAM* genes. The majority of transcripts code for CEACAMs with a short cytoplasmic tail lacking immunotyrosine-based motifs. No indication for GPI-anchored CEACAMs was found. Red stars indicate the presence of a stop codon, mutated splice sites are marked with a red tilde

Diversity of CEACAM proteins was found to be enhanced by differential splicing in various animal species. Since nothing is known about the splicing of equine *CEACAMs*, we designed primers which were predicted to allow the amplification of full length cDNAs (Table 3). Tissues and cells in which strong expression was detected were selected for the amplification of full length cDNAs. cDNAs were isolated from the gel, cloned into cloning vectors and sequenced. All identified CEACAM transcripts are schematically depicted in Fig. 2. *CEACAM1* was amplified from granulocytes and four different transcripts were identified. The extracellular part of the molecules contains an N-domain and 0, 1 or three IgC-like domains. Specifically, we have cloned CEACAM1-4 L, CEACAM1-2 L, CEACAM1-2S and CEACAM1-1 L. The long isoforms harbor an ITIM and an ITSM in its cytoplasmic tail. CEACAM43 was amplified from the cDNA of the kidney and only one transcript composed of 4 extracellular Ig domains and an ITIM/ITSM containing cytoplasmic tail (CEACAM43-4 L) was present. Full length transcripts of CEACAM41 in sufficient amount for further analysis were only found in activated PBMC. Two splice variants were identified differing by the content of sequence coding for the cytoplasmic tail. However, none of CEACAM41 transcripts contain the transmembrane exon sequence. Thus, both transcript variants code for the same protein, a secreted molecule without ITAM motif. Transcripts of *CEACAM42* were amplified from mRNA of the Caecum, since in different tissues from the small and large intestine a single full length transcript of around 1100 bp was amplified. The isolated transcript lacks sequences from the B-domain exon and from the C1 exon. Thus, CEACAM42 is a transmembrane molecule with two extracellular Ig-domains and a short cytoplasmic domain. *CEACAM45* transcripts contain the cytoplasmic C2 and C3 exons. However, these two exons do not belong to the coding sequence since a stop codon is located in the transmembrane domain which is included in both identified transcripts. The two transcripts of *CEACAM45* differ in their extracellular part; the major isoform has 3 Ig-domains while the minor isoform contains 2 Ig-domains. The transcript of *CEACAM50* did neither contain the B-domain exon nor the cytoplasmic exons C1 and C2. Thus CEACAM50 has also a short cytoplasmic tail. The transcript coding for CEACAM53 included all exons of the gene. *CEACAM54* has two unusual transcripts the first have an extended TM domain and the second did not include the TM domain.

**Table 3:** Gene-specific oligonucleotides for expression analyses and cDNA cloning of horse CEA gene family members

Gene	Oligonucleotide sequence	Location of primers (exon)	Size of PCR product (bp)
CEACAM1	For: TGCATCATATAAGATAGGCCCAG	N domain	367
	Rev: AGTGAGAGTCCTCTTGTCCAGG	A1 domain	
CEACAM41	For: CATTGCATGTGATAGAGCGAC	N domain	246
	Rev: CGTCCTTCTGTTCTGTGACTGT	A2 domain	
CEACAM42	For: AGGGGAAGGAATAGATCCCG	N domain	392
	Rev: GAGTCCTGTTGTCCGAGGATAG	A1 domain	
CEACAM43	For: CCCATCAAGAAATTGTGTCCT	N domain	276
	Rev: ATGTTAACACTACAGGGTCCCTG	A1 domain	
CEACAM44	For: GCTGTTGTAGGGACCGATGTTA	N domain	503
	Rev: CCTCCTTCCTGATGATGTGTGT	A2 domain	
CEACAM45	For: GCGATAGGGCAACAAGAAATTAT	N domain	254
	Rev: CATGTTAACACTACAGGGTCCTCA	A1 domain	
CEACAM46	For: AGTCCCACCCAATGGTATCC	N domain	525
	Rev: CCCAAGTATTGCCCCTTCTGT	A1 domain	
CEACAM47	For: ACAGACCAAGTCCCAAAACC	N domain	256
	Rev: TTACGTCTTTTGACGATTCCAG	A1 domain	
CEACAM48	For: GACCAGCTCGCAAACAAA	N domain	279
	Rev: AACAAGCTTCTTATCTGGCATTT	A2 domain	
CEACAM49	For: TGGAGCACGTCCACATAAAC	N domain	232
	Rev: GGAGGTATTTGACCCTGGATT	A2 domain	
CEACAM50	For: AGATGCTCTTGAAGGAACGGAT	N domain	453
	Rev: GACAGCTTCAGCCAGGTCCTA	A1 domain	
CEACAM51	ND	NA	NA
CEACAM52	For: ATGCTGCTGCAGGGGATA	N domain	508
	Rev: CATCCTCCCTCCTGACACAT	A2 domain	
CEACAM53	For: TTCAAAGGGGAAATAGATTCCA	N domain	394
	Rev: GAGTCCTGTTGTCTGGGGAC	A1 domain	
CEACAM54	For: ATCAGTCCCTGGCTTCAGA	N domain	409
	Rev: TACACGGAGCTGTATACTTC	A domain	
CEACAM55	For: CCCTACTAGTCACGAGGAAGAAC	N domain	222
	Rev: CATCCTCTCGGTCAGTCACA	A2 domain	
CEACAM1	For: GTCAGTAAGCTTCAGCAGACACCATGGAACTC	5’UTR	1624
	Rev: GTCAGTTCTAGACAGTGAACAGGGCAGGATATG	3’UTR	
CEACAM41	For: GAGCAGTGCTTGTGAGCATT	5’UTR	1131
	Rev: TAAGGGGAAGTTCCTGAAGG	3’UTR	
CEACAM42	For: TCACAGAGGGAGGGACAGAG	5’UTR	
	Rev: GGGTAAAGGGATCCTTCCAG	3’UTR	1390
	Rev: TCACAGAGGGAGGGACAGAG	TM domain	1121
CEACAM43	For: GTCAGTAAGCTTGACAGAGCAGGCAGCAGAC	5’UTR	1613
	Rev: GTCAGTTCTAGAGAACAGGGCAGGTTGCAT	3’UTR	
CEACAM45	For: TCACAGAGGAAAGGACAGAGC	5’UTR	
	Rev: CCTTGATTCCTGGACATTGAA	3’UTR	1539
	Rev: CCTTGATTCCTGGACATTGAA	TM domain	1379
CEACAM50	For: CAGGAGTGCTTGTGAGAGTT	5’UTR	1139
	Rev: AGGGAAGAGGCTTCGTCTTC	3’UTR	
CEACAM52	For: GTCAGTAAGCTTGCAGAAGCTCATCTCACAGAG	5’UTR	583
	Rev: GTCAGTTCTAGATTGTGAGGCAGATCAGATCC	3’UTR	
CEACAM53	For: CAGAGGGAGGGACAGAACAG	5’UTR	1163
	Rev: ATGGCAGTTAGCCTTGGAGA	3’UTR	
CEACAM54	For: TCTCACAGAGGGAGGAGCCA	5’UTR	916
	Rev: AGTCAGCAGTGCAGGAAACA	3’UTR	

*ND* not done, *NA* not applicable, *For* forward primer, *Rev.* reverse primer

### CEACAM42 has an extended transmembrane domain exon containing a stop codon

**Fig. 3:**
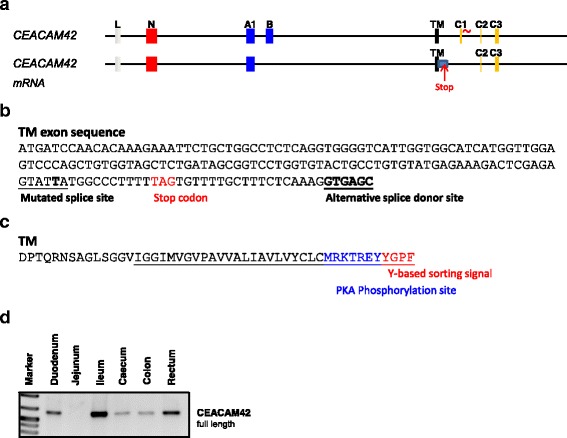
CEACAM42 has an extended transmembrane domain. The exon structure of the *CEACAM42* gene indicates that its gene product may contain an ITIM signaling motif. However as shown in **a** the cloned mRNA demonstrate that an alternative splice donor site of the transmembrane exon is used and that the exons C1 was not integrated into the mRNA. The stop codon which exists in the extended transmembrane exon is indicated by the red arrow and the “stop”. The mutated splice donor sites of C1 is indicated with a red tilde. **b** shows the possible but unused splice donor site (underlined). **c** Amino acid sequence of the transmembrane and cytoplasmic part of CEACAM42. The PKA phosphorylation site is highlighted in blue, the predicted Y-based sorting signal in red. **d** Expression of CEACAM42 was detected in various parts of the intestine

In *CEACAM42* the cytoplasmic exon 1 has a mutated splice donor site indicating that the cytoplasmic tail does not encode the ITIM/ITSM motifs. Indeed the structure of CEACAM42 mRNA demonstrated that the exon C1 is not included in the mRNA (Fig. 3a). Furthermore, there is also an alternative splice donor site at the end of the transmembrane domain exon. Thus, the transmembrane exon is extended by 37 nucleotides including a stop codon (Fig. 3b). Therefore the cytoplasmic exons 2 and 3 are not part of the coding sequence. The usage of the alternative splice donor site at the end of the transmembrane exon results in a transmembrane molecule with a short cytoplasmic tail of 11 amino acids. This short cytoplasmic tail contains a putative protein kinase A (PKA) phosphorylation site and a tyrosine-based sorting signal motif as predicted by the ELM software (Fig. 3c). In the intestine which is the main tissue of CEACAM42 expression (Table 1), only one transcript variant is expressed as indicated by a single band upon amplification of the full length cDNA (Fig. 3d).

### CEACAM50 has a unique signaling motif in the cytoplasmic tail

**Fig. 4:**
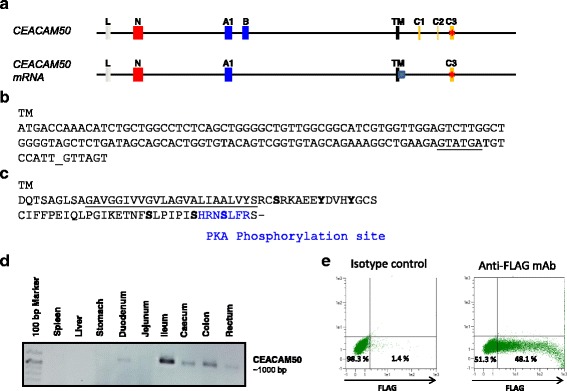
CEACAM50 has unique signaling motifs within the CEA gene family. The exon structure of the *CEACAM50* gene indicates that its gene product may contain an ITIM signaling motif. However as shown in **a** the cloned mRNA demonstrate that an alternative splice donor site of the transmembrane exon is used and that the exons C1 and C2 were not integrated into the mRNA. **b** shows the possible but unused splice donor site (underlined). **c** Amino acid sequence of the transmembrane and cytoplasmic part of CEACAM50. The PKA phosphorylation site is highlighted in blue, further phosphorylation sites were indicated in bold. **d** Expression of CEACAM50 was detected in various parts of the intestine. **e** Expression of CEACAM50 fused to a FLAG-tag at the N-terminus by transfected Cos7L. Red stars indicate the presence of a stop codon

*CEACAM50* has three cytoplasmic domain exons very similar to cytoplasmic exons of *CEACAM1*. Therefore, it may be expected that CEACAM50 has immune tyrosine-based signaling motifs. However, we found only transcripts lacking exon C1 and C2 (Fig. 4a). Furthermore, due to the use of an alternative splice donor site the transmembrane domain is prolonged by 14 nucleotides (Fig. 4b). Thus, the new splice donor site induces a frame shift and therefore the amino acid sequence of the cytoplasmic domain exon C3 is changed (Fig. 4b, c). Scanning the cytoplasmic tail of CEACAM50 for canonical signaling motifs identified a (PKA) phosphorylation site at the end of the cytoplasmic tail (Fig. 4c). Transcripts of *CEACAM50* were preferentially detected in tissues of the intestine (Fig. 4d). In order to confirm that CEACAM50 could be expressed at the cell surface we fused a FLAG-tag to the N-terminus of CEACAM50 and expressed the fusion protein in Cos7L cells. As shown in Fig. 4e the staining of living transfected cells with anti-Flag antibodies by flow cytometry strongly indicates that the fusion protein is located at the cell surface. Thus, CEACAM50 is a transmembrane CEACAM with a unique signaling motif not found in other species.

### CEACAM54 has a unique membrane proximal extracellular structure

**Fig. 5:**
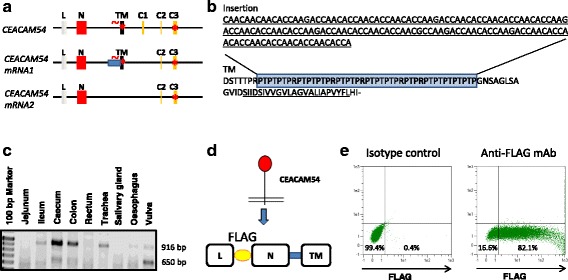
CEACAM54 contains a P-T-R rich membrane proximal extracellular region. **a** Exon structure of the *CEACAM54* gene and the two transcript variants identified in the present study. The simple sequence inserted in front of the transmembrane domain and the translation into the amino acid sequence (blue box) are depicted in (**b**). **c** Expression of transcript variant 1 (916 bp) in the intestine and trachea and of variant 2 (650 bp) in the vulva mucosa. Cartoon of the Flag-tag-CEACAM54 fusion protein (**d**). Expression of the Flag-tagged CEACAM54 at the cell surface of Cos7L cells as detected by flow cytometry (**e**). Red stars indicate the presence of a stop codon, mutated splice sites are marked with a red tilde

The *CEACAM54* gene consists of six exons (Fig. 5a), the transmembrane exon and the C3 exon contains stop codons. The canonical splice acceptor site of the transmembrane exon is disrupted, due to the insertion of a simple sequence repeat (SSR) (Fig. 5a and b). An alternative splice acceptor site is located in front of the inserted simple sequence repeat. Indeed as demonstrated by cDNA cloning, two transcript variants were identified. The first variant contains the transmembrane exon including the SSR, whereas in the second transcript the transmembrane exon, including the SSR, and the C1 exon are excluded (Fig. 5a). The first transcript variant has an open reading frame until the stop codon at the end of the transmembrane exon. The SSR codes for a Proline, Threonine and Arginine-rich extracellular membrane proximal region. CEACAM54 mRNA was detected in the intestine, trachea and vulva. Interestingly, while in most tissues transcript variant 1 was dominant, in the vulva mucosa variant 2 was prominent (Fig. 5c). Again we tested if the mRNA is translated into protein and if this protein is expressed at the cell surface. As shown in Fig. 5d and e Flag-tagged CEACAM54 is expressed at the cell surface of transfected Cos7L cells.

### Loss of membrane-anchorage and signaling via an ITAM of CEACAM41

**Fig. 6:**
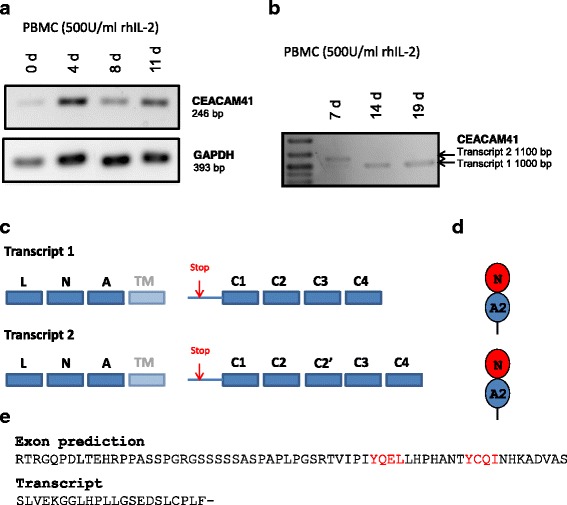
CEACAM41 is a secreted protein. **a** PBMC were cultured with 500 U/ml rhIL-2 for the indicated times. RT-PCR using primers located in the leader and the N-domain suggested that CEACAM41 is preferentially expressed by stimulated PBMC. Full length amplification of CEACAM41 cDNA identified two different transcripts (**b**). The two transcripts differ from each other by a short sequence, inserted between exon C2 and C3 further named C2’ exon (**c**). The stop codon which exists in the extended C1 exon is indicated by the red arrow and the “stop” (**c**). Both transcripts code for the same protein (**d**). **e** Comparison of the amino acid sequence of the cytoplasmic tail of CEACAM41 as predicted from the exon sequence of the CEACAM41 gene and the peptide sequence encoded by the cytoplasmic exons in transcripts without transmembrane domains

*CEACAM41* gene is the only equine CEACAM that harbors exons which may code for an ITAM in the cytoplasmic tail similar to that found in human *CEACAM3* and *CEACAM4*. In humans, ITAM harboring CEACAMs are expressed specifically in granulocytes [18, 19]. However, we did found CEACAM41 transcripts only in granulocytes isolated from one out of three horses. In addition, we did not find CEACAM41 transcripts in any other tissue we have analyzed. Since expression of some CEACAMs, i.e. CEACAM1 by T cells is activation dependent we analyzed expression of CEACAM41 by stimulated equine PBMC (Fig. 6a). Amplification of full length CEACAM41 cDNA reveals that two different transcripts are generated in IL-2 activated PBMC (Fig. 6b). Cloning and sequencing of both cDNAs demonstrated that in both transcripts the transmembrane exon is excluded leading to a frame shift and a new stop codon at the 5′-end of the extended C1 exon (Fig. 6c). Thus, both transcripts code for one protein which consist of one IgV-like and one IgC-like domain followed by a short peptide (Fig. 6d). The short peptide has no sequence similarity to the predicted cytoplasmic sequence of the genomic exon sequence containing an ITAM-like motif (Fig. 6e).

## Discussion

Gene duplication (GD) and alternative splicing (AS) are the two main mechanisms responsible for functional protein diversity [20, 21]. GD generates a significant expansion of the CEA gene family in various mammalian species resulting in up to 100 different *CEACAM* genes in certain bat species [9]. Protein diversity in the CEA family is further enhanced by extensive AS of certain CEACAM mRNAs, for example human *CEACAM1* codes for 12 different proteins [22, 23]. A more sophisticated interplay between GD and AS is the possibility to generate functional diversity of duplicated genes by AS [24]. Variation of AS between the ancestral gene and the duplicated gene may favor the fixation of the duplicate, due to new functional properties gained by AS. Indeed, it was observed that duplicated genes have a reduced amount of AS events per gene compared with the ancestral gene [24]. Such a reduction of AS in duplicated genes may change their function in the context of a specific cell type or tissue were they are expressed. Consistent with this hypothesis we only found a single splice isoform for CEACAM43 while four isoforms of CEACAM1 were detected. Surprisingly, AS was not found to play a major role for functional diversification of *CEACAM1* paralogs in CEA gene families so far investigated in more detail. However, the discrepancy between the predicted structure of equine *CEACAMs* and the sequenced mRNAs suggests that AS is a pivotal mechanism for *CEACAM1* paralog diversification in the horse and most likely in other mammals. However, we would like to point out that the absence of finding particular splice variants, does not mean that these protein variants are not expressed anywhere in the horse tissues, since both tissue distribution and cellular activation state are known to influence processing of the transcripts from the CEA family. AS effects primarily the transmembrane domain exon, either at the 5′ end or the 3′ end. The reason may be that modifications at the transmembrane domain have a high probability to change the function of the duplicated gene product by means of modifying signal transduction [25, 26]. Four out of seven equine CEACAM1 paralogs have gained new signaling motifs by AS. For example, CEACAM42 and CEACAM50 mRNA were modified by using an alternative splice donor site at the transmembrane exon and to ignore splice acceptor sites of the first and second (only CEACAM50) cytoplasmic exons. In both cases the potential ITMS/ITIM motifs were eliminated. An AS of CEACAM54 mRNA leads to two transcripts, one having a very unique proline-threonine-arginine-rich domain encoded by a SSR at the membrane proximal part of the extra cellular region, and the second missing a transmembrane region. Since the P-T-R-rich domain is not found in any other mammalian protein in the NCBI-data bases we argued that this protein could be expressed. However, we were able to express FLAG-tagged CEACAM54 on the cell surface of Cos7 cells by transient transfection, indicating that CEACAM54 is expressed in the horse. Taken together, only CEACAM1 and CEACAM43 retained the inhibitory signaling motifs. This is similar to the CEA gene family in mice which also contain two inhibitory CEACAMs [27]. And also the different expression pattern of inhibitory CEACAMs was previously observed for murine CEACAM1 and murine CEACAM2 [10].

One reason for the restricted number of inhibitory CEACAMs may be that CEACAM1 is a checkpoint molecule for T and B cell activation, and therefore an increase of the gene dosage, after GD, may be fatal to a well-balanced regulation of immune responses [28]. Similar observations were made in the Signaling regulatory protein (SIRP) family. Although the founder gene is the inhibitory receptor SIRPα [29], the expanded SIRP families comprise only a single ITIM containing receptor in all investigated species [30]. The authors suggested that this might be consistent with a homeostatic function of SIRPα, like recognizing “self” in the form of the broadly expressed surface marker CD47, which negatively regulate the function of innate immune cells, such as macrophages [30]. The number of activation receptors is not limited like the inhibitor receptors in families containing paired receptors like the CEA, SIRP and natural killer cell inhibitory receptor (KIR) gene families [13, 30, 31].

Furthermore, and in certain aspects the most striking modification induced by AS was observed for CEACAM41 mRNA. Both transcripts code for the same amino acid sequence lacking a transmembrane domain. The GPI anchored CEACAMs in primates have a stop codon within the transmembrane domain which shortens the transmembrane helix in the way that they do no longer have a charged amino acid at the cytoplasmic border of the cell membrane, thus it is not properly fixed in the cell membrane. On the other hand the residual transmembrane domains provide the necessary signals for the GPI anchorage. In the horse the secreted CEACAM41 did not contain any part of the transmembrane domain, thus also lacking a GPI signal. This indicates that CEACAM41 is not attached to the cell membrane, but it is secreted. Thus no activating CEACAM exists in the horse. This is again similar to the murine CEA family which does not contain activating CEACAMs [32]. From an evolutionary point of view, the presence of a putative activating CEACAM receptor gene which still has intact exons and splice sites indicates that a functional activating receptor in the horse existed until recently. One may speculate that at a certain point of equine history the selective pressure, putatively a pathogen, for the activating CEACAM got lost. From that time point on the activating CEACAM was free to change his function or to get eliminated from the genome. AS which renders the activating receptor into a secreted molecule would change the function of the molecule both rapidly and fundamentally. Once a completely new CEACAM is created selection may search for an optimized spatio-temporal expression pattern for the new function. This period of selection may have led to the expression of CEACAM41 by activated PBMC. Remarkably, CEACAM41 is not the only secreted CEACAM expressed by equine activated PBMC, since CEACAM46a and CEACAM46b are also secreted by PBMC upon activation [15]. Comparing the ligand binding domain (N-domain) of other secreted equine CEACAMs showed that CEACAM44 and CEACAM55 cluster together with that of CEACAM46a and CEACAM46b suggesting that they may share common ligands [15]. It is well known that hemophilic interaction of membrane bound CEACAMs regulate the activation of immune cells in trans [33, 34]. Furthermore it was described that soluble CEACAMs interact with membrane-bound CEACAMs in a homophilic and heterophilic fashion [35–37]. Thus it is tempting to speculate that equine lymphoid cells secrete CEACAMs upon activation in order to transmit regulatory signals to distantly located immune cells. Putative ligands of the secreted CEACAMs on immune cells may include CEACAM1, CEACAM43 and CEACAM45. Together these considerations suggest that equine lymphoid cells have acquired a novel mechanism based on the secretion of CEACAMs to regulate an immune response. Further investigations are required to substantiate secreted CEACAMs could be useful therapeutic targets to modulate immune responses in the horse.

## Conclusion

Gene family expansion is a potent evolutionary process to adapt to environmental cues. In most cases gene duplication is accompanied by sequence diversification of paralogues genes. Recently we have identified that in certain bat species the ligand-binding domain of CEACAMs is under positive selection. In the present report we show, that in the horse a second mechanism of gene diversification is active. AS after gene duplication, that preferentially affect cell membrane anchorage and the cytoplasmic tail of CEACAM1 paralogs, is most likely a mechanism that may rapidly change the functional properties of the paralogous gene by changing its signaling capacity. Such potent mechanisms of gene variation may extraordinarily accelerate adaption to environmental cues. It is intriguing that such a mechanism is involved in the evolution of a gene family which is thought to be part of host-pathogens arms race.

## Methods

### Cells and tissues

Different equine tissue samples were collected from freshly slaughtered healthy horses and either flash-frozen in liquid nitrogen or stored in RNAlater (Invitrogen). Peripheral blood mononuclear cells (PBMCs) and granulocytes were isolated from blood of healthy horses by density-gradient centrifugation through Ficoll-Paque 1077 g/l (GE Healthcare). Stimulation of PBMC with human rIL-2 (Proleukin, Chiron) was performed with 200 U/ml for the indicated time, at a concentration of 5 × 10^5^ cells/ml in RPMI-1640 supplemented with 10% fetal calf serum (FCS “Gold”; Bio&SELL), 2 mM L-glutamine, 100 U/ml penicillin, 100 μg/ml streptomycin, non-essential amino acids and 1 mM sodium pyruvate (GIBCO/Invitrogen). Magnetic cell separation (Miltenyi Biotec) was used for the isolation of lymphocyte subtypes. CD4 and CD8 positive cells were isolated with murine IgG1 primary mAb (compare “Cell transfection and flow cytometry” below) and anti-mouse IgG MicroBeads.

### Identification and prediction of equine CEACAMs

Equine CEACAMs were identified similar to the method described previously [9]. For sequence similarity searches we used the NCBI BLAST tools “blastn” http://blast.ncbi.nlm.nih.gov/Blast.cgi and Ensembl BLAST/BLAT search programs http://www.ensembl.org/Multi/Tools/Blast?db=core using default parameters. For identification of horse *CEACAM* exons, exons from known *CEACAM* and *PSG* genes from other species were used to search “whole-genome shotgun contigs (wgs)” databases limited to organism “*Equus caballus* (taxid:9796)”. Hits were considered to be significant if the E-value was < e-10 and the query coverage was > 50%. Once a wgs contig containing CEACAM-related sequences was identified we manually confirmed the presence of the complete exon according to its size and the presences of CEACAM-typical splice site sequences. The gene structure was predicted according to known CEACAMs. Predicted CEACAM genes were further compared with the horse genome Ensembl/NCBI release EquCab2.

### Expression analysis by reverse transcription-polymerase chain reaction

RT-PCR was carried out as previously described [2]. In brief, total RNA was extracted using the RNeasy kit (Qiagen). One microgram of total RNA was transcribed using AMV Reverse Transcriptase (Promega). The RT product was amplified with Taq polymerase (Fermentas). After denaturation at 95 °C for 45 s, 35 PCR cycles (denaturation: 95 °C, 30 s; annealing: 60 °C, 1 min; extension: 72 °C, 1.5 min) and a final extension at 72 °C for 15 min were performed. Primers used were summarized in Table 3. Eight microliters of each PCR product were analyzed by electrophoresis on a 1.8% agarose gel and visualized by GelRed (Biotium) staining.

### cDNA cloning

RNA isolation an RT was performed as described for expression analysis. Primers used for amplification of a full-length cDNAs are shown in Table 3. For cDNA cloning the RT product was amplified by polymerase chain reaction (PCR) with Easy-A High-Fidelity PCR Cloning Enzyme (Agilent) and analyzed by agarose gel electrophoresis. Specific bands were extracted from the agarose gel using QIAEX II Gel Extraction Kit (Qiagen). The PCR-products were cloned using the StrataClone PCR Cloning Kit (Agilent). Plasmid DNA isolated from various clones were analyzed by PCR and sequencing. Nucleotide sequencing was performed with the BigDye Terminator Cycle Sequencing Kit (PE Applied Biosystems).

### Cell transfection and flow cytometry

For expression of equine CEACAMs by eukaryotic cells, full length cDNA was transferred from the StrataClone Cloning vector into the shuttle vector pFLAG-CMV3 (Sigma-Aldrich). 1 × 10^6^ Cos7 cells were transfected using the Nucleofector Kit V (Amaxa). 1 × 10^5^ transiently transfected Cos7 cells were stained with murine anti-FLAG mAb (clone M2, Sigma-Aldrich) as a primary mAb and using anti-mouse IgG-PE as secondary antibody (goat anti-mouse). Flow cytometry was performed with the MACSQuant Analyzer and the “MACS Quantify” software.

### Bioinformatics

Phylogenetic analyses based on nucleotide and amino acid sequences were conducted using MEGA6. Sequences were aligned using “Muscle” and the maximum likelihood (ML) or unweighted pair group method with arithmetic mean (UPGMA) method with bootstrap testing (500 replicates) was applied for the construction of phylogenetic trees. Sequence motif identification was performed using the sequence pattern search program ELM (http://elm.eu.org/). Transmembrane helixes were identified using the TMHMM Server at http://www.cbs.dtu.dk/services/TMHMM-2.0/ and GPI anchors were predicted by the PredGPI Server at http://gpcr.biocomp.unibo.it/predgpi/. Phosphorylation sites were identified by the NetPhos 3.1 Server at http://www.cbs.dtu.dk/services/NetPhos/.

## Abbreviations

AS: Alternative splicing
CEA: Carcinoembryonic Antigen
CEACAM: Carcinoembryonic Antigen-related cell adhesion molecule
GD: Gene duplication
GPI: Glycosylphosphatidylinositol
ISF: Immunoglobulin super family
ITAM: Immunoreceptor tyrosine-based activation motif
ITIM: Immunoreceptor tyrosine-based inhibitory motif
ITSM: Immunoreceptor tyrosine-based switch motif
KIR: Killer cell inhibitory receptor
ML: Maximum likelihood
PBMC: Peripheral blood mononuclear cells
PCR: Polymerase chain reaction
PSG: Pregnancy-specific glycoproteins
RT: Reverse transcription
SIRP: Signaling regulatory protein
TM: Transmembrane
WGS: Whole-genome shotgun
